# Effectiveness of Flexible Ureterorenoscopy and Laser Lithotripsy for Multiple Unilateral Intrarenal Stones Smaller Than 2 cm

**DOI:** 10.1155/2014/314954

**Published:** 2014-06-12

**Authors:** Erdal Alkan, Oguz Ozkanli, Egemen Avci, Mirac Turan, M. Murad Başar, Oguz Acar, M. Derya Balbay

**Affiliations:** ^1^Department of Urology, Memorial Sisli Hospital, Okmeydanı, Şişli, 34385 Istanbul, Turkey; ^2^Department of Anesthesiology, Memorial Sisli Hospital, Okmeydanı, Şişli, 34385 Istanbul, Turkey; ^3^Department of Urology, Memorial Atasehir Hospital, Atasehir, 34758 Istanbul, Turkey

## Abstract

*Purpose*. To evaluate the safety and efficacy of RIRS for the treatment of multiple unilateral intrarenal stones smaller than 20 mm. *Methods*. Between March 2007 and April 2013, patients with multiple intrarenal stones smaller than 20 mm were treated with RIRS and evaluated retrospectively. Each patient was evaluated for stone number, stone burden (cumulative stone length), operative time, SFRs, and complications. *Results*. 173 intrarenal stones in 48 patients were included. Mean age, mean number of stones per patient, mean stone burden, and mean operative time were 40.2 ± 10.9 years (23–63), 3.6 ± 3.0 (2–18), 22.2 ± 8.4 mm (12–45), and 60.3 ± 22.0 minutes (30–130), respectively. The overall SFR was 91.7%. SFRs for patients with a stone burden less and greater than 20 mm were 100% (23/23) and 84% (21/25), respectively (*χ* ^2^ = 26.022, *P* < 0.001). Complications occurred in six (12.5%–6/48) patients, including urinary tract infection or high-grade fever >38.5°C in three cases, prolonged hematuria in two cases, and ureteral perforation in one case, all of whom were treated conservatively. No major complications occurred. *Conclusions*. RIRS is an effective treatment option in patients with multiple unilateral intrarenal stones especially when the total stone burden is less than 20 mm.

## 1. Introduction

According to the European Association of Urology (EAU) guidelines, extracorporeal shock wave lithotripsy (ESWL) for the management of the renal stones <20 mm is recommended as a first line treatment [1]. For these stones, the success rate of ESWL has ranged from 37% to 90% depending on several factors [2–4]. Multiple stones are found in 20–25% of patients with urolithiasis [3, 5, 6]. When multiple renal stones are treated with ESWL, the success rate decreases to 50%, especially for lower calyx stones [1, 2]. Thus there has not been any consensus about the management for multiple intrarenal stones in both the EAU and the American Urological Association (AUA) guidelines [1, 2].

With the advancement in endoscopic technology, a new dimension has been opened in the treatment of stone disease. Entire urinary collecting system either unilaterally or bilaterally can be reached using flexible ureteroscope; stones can be actively fragmented via holmium laser and removed by some special basket catheters. The indication of flexible ureteroscopy has been extending, including intrarenal stones, ESWL failure, morbid obesity, musculoskeletal deformities, bleeding diathesis, and occupations that require complete stone clearance (i.e., pilots) [7–9]. Retrograde intrarenal surgery (RIRS) is likely to be an ideal treatment for multiple unilateral intrarenal stones that are smaller than 20 mm, with a high stone-free rate and low morbidity, and may represent a possible alternative to ESWL [10]. Herein, we present our experience using flexible ureteroscopy and/or holmium laser lithotripsy for treating multiple unilateral intrarenal stones smaller than 20 mm.

## 2. Material and Methods

Between March 2007 and April 2013, patients with multiple (at least 2 stones) unilateral intrarenal stones smaller than 20 mm were treated with RIRS and evaluated retrospectively. Forty-eight patients (37 males and 11 females) were treated with flexible ureteroscope for multiple intrarenal stones and met the selection criteria. The mean age and mean body mass index (BMI) were 40.2 ± 10.9 years (23–63) and 29.2 ± 5.1 kg/m^2^ (21–44), respectively. All patients were evaluated by computed tomography (CT) scan with stone protocol prior to the procedure. Each patient was evaluated for stone location, stone number, stone size, stone burden (cumulative stone length of the intrarenal stones), BMI, operative time, stone-free rates (SFRs), and perioperative complications. Total stone burden was calculated as the sum of each stone size. Stone burden was classified as 20 mm or less and greater than 20 mm. Multiple intrarenal stones in patients in whom ESWL or percutaneous nephrolithotomy (PCNL) had failed, patients' characteristics (i.e., obesity, coagulopathy, or solitary kidney), and preferences were the indications for RIRS in the present study. Prior to the procedure, informed consent was obtained from all patients. Patients were followed up for at least 3 months after surgery to evaluate any complications and assess the SFR.

At the start of the operation, intravenous antibiotic prophylaxis with second-generation cephalosporin was administered unless urine culture suggested any different antibiotic. All operations were performed using URF P-5 flexible ureteroscope (Olympus, Tokyo, Japan) and Cobra Flexible Dual-Channel Ureteroscope (Richard Wolf, Knittlingen, Germany), according to its availability, through a ureteral access sheath (Flexor ureteral access sheath 12/14F 35 cm; Cook Medical, Bloomington, IN, USA) with a pressurizing irrigation system. In case the ureteral access sheath could not be advanced due to ureteral stricture, a DJS was inserted into the ureter and the intervention was delayed at least 15 days. Holmium YAG laser (Sphinx, Lisa Laser, 30 watts, Katlenburg, Germany) in combination with 200 *μ*m (LithoFib, Lisa Laser, Katlenburg, Germany) or 272 *μ*m (FlexiFib, Lisa Laser, Katlenburg, Germany) laser fibers was set at an energy level of 0.6–1.4 J and at a rate of 5–8 Hz, if lithotripsy was necessary. A nitinol basket (Ngage nitinol stone extractor 2,2F 115 cm basket; Cook Medical, Bloomington, IN, USA) was used for the removal of stone fragments. Endoscopically, intraoperative success was defined as extraction of all stone fragments or laser lithotripsy of all stones to less than 4 mm fragments. At the end of the procedure, a 4.8F 26 cm double J stent (DJS) was inserted based on surgeon decision and the DJS was removed under local anesthesia using flexible cystoscope within 4 weeks postoperatively. Residual fragments were assessed with noncontrast CT or urinary ultrasound (US) two months after DJS removal. Based on the EAU guidelines 2014, stone-free status was defined as the absence of stone fragments or asymptomatic insignificant residual fragments of <4 mm (1). Perioperative complications were recorded.

For statistical calculations, Statistical Package for Social Sciences, version 15.0 (SPSS, Inc., Chicago, IL, USA), was used. The arithmetic mean and standard deviation of the data were determined. The Pearson chi-square test was used to compare the proportion of stone-free rates and complications. Statistical significance was accepted at *P* < 0.05.

## 3. Results

The patients' stone demographics are shown in Table 1. RIRS was performed in 39 (81.2%) patients as a primary procedure. Additionally it was performed after failed ESWL in 7 (14.6%) and failed PCNL in 2 (4.2%) cases. Flank pain was the most important indication of RIRS, which occurred in the 43.7% of the patients. Other indications of RIRS were recurrent urinary infection (6.3%), hematuria (6.3%), patients' characteristics (8.3%; two patients were morbidly obese, and two patients had a solitary kidney), and preferences (35.4%). Intraoperative and postoperative features of the patients are summarized in Table 2. Holmium laser was used in 45 of 48 patients (93.8%); nonetheless, in the remaining 3 cases (6.2%), one of them was a pilot, who had stones smaller than 5 mm, only nitinol basket was used. Basket catheter was used in 30 patients (62.5%) after stone fragmentation. Stone fragments were not removed after holmium laser lithotripsy in the remaining 18 patients (37.5%) who had heavy stone burden. Stone-free status was evaluated by urinary system CT at postoperative 3 months, except for 25 patients (52%) who were rendered stone-free according to the intraoperative findings. These patients were followed up by ultrasonography. At the end of the procedure, 4.8 F 26 cm DJS was placed in 33 patients (68.8%) (Table 2).

Twenty-three patients (48%) had a stone burden <20 mm, and 25 patients (52%) had a stone burden ≥20 mm. The overall SFR was 91.7%. SFRs for patients with a stone burden less and greater than 20 mm were 100% (23/23) and 84% (21/25), respectively (*χ* ^2^ = 26.022, *P* < 0.001) (Tables 1 and 2). There were no residual stone fragments in 25 (52.1%) patients (13 patients with stone burden <20 mm; 12 patients with stone burden ≥20 mm); nonetheless there were clinically insignificant residual fragments (CIRF) in 19 (39.6%) patients (10 patients with stone burden <20 mm; 9 patients with stone burden ≥20 mm). A total of four patients (8.3%), all of whom had a stone burden ≥20 mm, had residual stone fragments ≥4 mm after surgery. Of these four patients, two patients had a 5 mm fragment in the lower calyx and 4 mm fragment in the middle calyx, one patient had a 6 mm fragment in the lower calyx and two 3 mm fragments in upper calyx, and one patient had a 7 mm fragment after the treatment. These patients were offered follow-up surgery or ESWL but all refused since they remained asymptomatic postoperatively.

There were no major perioperative complications. Minor complications were recorded in six (12.5%–6/48) patients, including urinary tract infection or high-grade fever >38.5°C in three cases, prolonged hematuria lasted longer than a week in two cases, and minor ureteral perforation in one case. The patients who had urinary tract infection were treated with appropriate oral antibiotics in outpatient setting. Patients with prolonged hematuria were treated conservatively and cured without any transfusion. Intraoperatively minor ureteral perforation was seen in one patient, but the procedure was completed without any difficulties. A DJS was kept in this patient for 2 months. A follow-up CT at 1 month after DJS removal showed ureteral healing without any complication. No episode of renal colic was seen in the follow-up period in patients with stone fragments >4 mm after RIRS.

## 4. Discussion

There has not been any consensus about the management for multiple unilateral intrarenal stones in both the EAU and the AUA guidelines [1, 2]. According to the EAU and AUA guidelines, ESWL achieves excellent SFRs for stones up to 20 mm and is recommended as a first line treatment for such stones [1, 2]. The SFRs for single stones in the ureteropelvic junction or renal pelvis are 80%–88% [11]. However, SFRs are lower in the single calyx stones, which are 73%, 69%, and 63% for upper, middle, and lower calyxes, respectively [9, 11, 12]. When multiple stones are present, the SFR of ESWL drops down to 50% to 55%, especially for lower calyx stones [1, 2, 11, 13]. A study by Abe et al. reported that the SFR was just 41% for multiple stones, but the SFR was 71% for single renal stone [5]. Short convalescence, patient acceptance, and the lack of a requirement of anesthesia during the procedure are some of the advantages of ESWL [11, 12]. However, there are many factors including patient characteristics (i.e., body mass index) and stone characteristics, such as stone number, size, localization (i.e., lower calyx), and composition (i.e., calcium oxalate monohydrate), in which the effectiveness of ESWL is limited [2, 5, 13]. Besides, ESWL is contraindicated in patients with coagulopathy and in pregnancy [2].

Along with the technological advancements, the use of flexible ureteroscope combined with holmium laser lithotriptors has increased in the treatment of intrarenal stones and has made RIRS an alternative to ESWL and PNL for treating renal stones. There are several advantages of RIRS over ESWL. First, due to detailed intrarenal examination, anatomical problems could be detected and treated endoscopically. Second, flexible ureteroscopic lithotripsy allows active fragmentation of all stone types, and also stones could be fragmented down to tiny gravel particles. Third, relatively fragmented large particles especially in the lower calyx could be extracted using nitinol baskets through the access sheath. Lastly, RIRS compared with ESWL has an excellent SFR in the management of multiple intrarenal stones in particular less than 20 mm stones [10, 14]. Conversely, RIRS has some disadvantages such as requirement of anesthesia and its being an invasive procedure.

There are some studies on RIRS of multiple unilateral intrarenal stones [9, 10, 14–16]. In a study by Breda et al., there were 24 patients with stone burden ≤20 mm and 27 patients with stone burden >20 mm [10]. In their series SFR after two procedures was 100% and 85%, respectively. In another study of Takazawa et al., 51 patients with multiple renal stones who had undergone RIRS were evaluated [14]. In patients with stone burden <20 mm and ≥20 mm, SFRs after one and two sessions were 92% and 100% versus 69% and 85%, respectively. SFR was accepted when residual fragment <2 mm. In our study, the overall SFR was 91.7%. SFRs for patients with a stone burden less and greater than 20 mm were 100% (23/23) and 84% (21/25), respectively (*P* < 0.001). These results were statistically significant and compatible with the literature. Our SFR after a single session is equivalent to the SFR reported in the previous studies after two RIRS procedures for two reasons: 1) we have excluded patient with stone size greater than 20 mm 2) SFR in this study defined as either complete absence of any residuals or presence of fragments <4 mm.

The combination of flexible ureteroscopes, laser lithotripsy, and nitinol stone baskets provides excellent SFRs with low postoperative complication in the management of renal stones [10, 17]. In our study, while holmium laser and basket catheter were used in 27 (56.3%) cases, holmium laser without basket catheter was used in 18 (37.5%) cases that had heavy stone burden. Although the usefulness of stone basket has been reported in the literature, we did not use any basket catheter in patients where efficacious fragmentation down to <4 mm in size was done by visual assessment in order not to prolong the procedure [10, 17].

The majority of stones were located in lower calyx in patients with multiple renal stones [18]. Due to technical difficulties in accessing lower calyceal stones, the treatments of these stones are much difficult compared to stones located in other calyxes. In a study by Jung et al., it was shown that SFR was 81% for the management of ESWL resistant stones located in lower calyx by RIRS [19]. RIRS is a better choice than PCNL given the lower complication rates of RIRS for lower calyx stones are measuring 1-2 cm [20]. In our study, a total of 69 (40.1%) stones were located in the lower calyx. These stones were relocated by nitinol basket, carried into upper or middle calyx, and effectively fragmented by holmium laser. Our results are compatible with the literature.

EAU guidelines on the management of renal stones recommended PCNL as the first line treatment for stones greater than 20 mm [1]. Nonetheless PCNL is also recommended for lower calyx stones greater than 10 mm [1]. Although PCNL is more effective treatment option than RIRS or ESWL in the management of renal stones, it has serious complications such as bleeding and urine leakage [21]. With the development of the smaller sheaths, mini-PNL (miniperc) and micro-PNL (microperc) could be performed with the minimal complication rates, and these techniques are used in the treatment of small intrarenal stones [22]. The reported SFR with micro-PNL ranges from 85% to 93%, which is similar to RIRS [22, 23]. In a study by Piskin et al., which included 11 patients with a mean stone size of 12.8 mm (7–18), the SFR was 85% [23]. Patients presented in our series could have been treated with miniperc or microperc as well. However, since we are not experienced in any of these techniques we opted to treat patients with RIRS.

Recent reports indicate that the overall complication rates of ureteroscopic procedures are between 6% and 16% [24, 25]. The most encountered complications are urinary tract infections, minor ureteral injuries, hematuria, and postoperative renal colic [10, 24, 25]. In our study, all complications encountered were minor and seen in only 6 (12.5%) of our patients. Three patients had urinary tract infection and were treated with appropriate antibiotics. Two patients had prolonged hematuria and were treated with conservatively. Finally one patient who had minor ureteral perforation was treated with DJS placement. Overall, our complications were similar to the literature.

CT and US are the most commonly used instruments in the diagnosis of urinary system stones. The sensitivity and specificity of CT for diagnosing renal stones have been reported as 95%, 96%, and 98% to 100%, respectively [26]. On the other hand, the sensitivity of US for detecting renal stones has been variably reported to be between 12% and 98% [27]. Although US is highly effective in showing stones larger than 5 mm, its effectiveness in the diagnosis of stones smaller than 3 mm is poor [27]. In our study, CT and urinary US were performed in 23 and 25 patients, respectively. Twenty-five patients who underwent US at third postoperative month were rendered stone-free already at the time of surgery as per intraoperative findings.

There are some limitations to our study. First, it was a retrospective review from a single center. Second, the study had small number of patients. Larger series with longer follow-up are necessary to confirm the long-term value in the treatment of multiple intrarenal stones. Third, all cases were done by three surgeons. Lastly, stone clearance rates were not assessed by the same technique. In spite of these limitations, our study shows that RIRS is an effective treatment modality with high SFR and minor complications in the management of multiple intrarenal stones smaller than 2 cm.

## 5. Conclusions

RIRS is an effective treatment option in patients with multiple unilateral intrarenal stones especially when the total stone burden is less than 20 mm, and it is associated with high success and low complication rates. In order to recommend RIRS on the first line treatment, stronger studies with comparative data are needed.

## Figures and Tables

**Table 1:** Preoperative stone demographics.

Total stone number	173
Mean stone number	3.6 ± 3.0 (2–18)
Mean stone size (mm)	6.6 ± 3.6 (2–17)
Mean stone burden (mm)	22.2 ± 8.4 (12–45)
<20 mm (*n* = 23)	16.1 ± 2.4 (12–19)
≥20 mm (*n* = 25)	27.8 ± 8.1 (20–45)
Localization (*n*)	
Upper calyx	21 (12.1%)
Middle calyx	42 (24.2%)
Lower calyx	69 (40.1%)
Renal pelvis	41 (23.6%)
Lateralization	
Right side	23 (48%)
Left side	25 (52%)

**Table 2:** Evaluation of intraoperative and postoperative data.

Mean operative time (min)	60.3 ± 22.0 (30–130)
Use of holmium laser only (*n*)	18 (37.5%)
Use of basket catheter only (*n*)	3 (6.2%)
Use of laser and basket catheter (*n*)	27 (56.3%)
DJS placement (*n*)	33 (68.8%)
Mean duration of DJS (day)	27.8 ± 8.3 (7–60)
Mean hospital stay (hour)	24.1 ± 3.8 (18–48)
Overall SFR (%)	91.7
Stone burden <20 mm	100
Stone burden ≥20 mm	84
Complications (*n*)	6 (12.5%)
UTI	3
Prolonged hematuria	2
Ureteral perforation	1
